# Reverse U-to-C editing exceeds C-to-U RNA editing in some ferns – a monilophyte-wide comparison of chloroplast and mitochondrial RNA editing suggests independent evolution of the two processes in both organelles

**DOI:** 10.1186/s12862-016-0707-z

**Published:** 2016-06-21

**Authors:** Nils Knie, Felix Grewe, Simon Fischer, Volker Knoop

**Affiliations:** Abteilung Molekulare Evolution, IZMB – Institut für Zelluläre und Molekulare Botanik, Universität Bonn, Kirschallee 1, D-53115 Bonn, Germany; Present address: Department of Science and Education, Field Museum of Natural History, Integrative Research Center, 1400 South Lake Shore Drive, Chicago, IL 60605 USA; Present address: Protrans medizinisch diagnostische Produkte GmbH, Ketschau 2, D-68766 Hockenheim, Germany

**Keywords:** RNA editing, Ferns, *Equisetum*, PPR proteins, Reverse editing, Monilophytes, Mitochondria, Chloroplasts, Editing loss

## Abstract

**Background:**

RNA editing by C-to-U conversions is nearly omnipresent in land plant chloroplasts and mitochondria, where it mainly serves to reconstitute conserved codon identities in the organelle mRNAs. Reverse U-to-C RNA editing in contrast appears to be restricted to hornworts, some lycophytes, and ferns (monilophytes). A well-resolved monilophyte phylogeny has recently emerged and now allows to trace the side-by-side evolution of both types of pyrimidine exchange editing in the two endosymbiotic organelles.

**Results:**

Our study of RNA editing in four selected mitochondrial genes show a wide spectrum of divergent RNA editing frequencies including a dominance of U-to-C over the canonical C-to-U editing in some taxa like the order Schizaeales. We find that silent RNA editing leaving encoded amino acids unchanged is highly biased with more than ten-fold amounts of silent C-to-U over U-to-C edits. In full contrast to flowering plants, RNA editing frequencies are low in early-branching monilophyte lineages but increase in later emerging clades. Moreover, while editing rates in the two organelles are usually correlated, we observe uncoupled evolution of editing frequencies in fern mitochondria and chloroplasts. Most mitochondrial RNA editing sites are shared between the recently emerging fern orders whereas chloroplast editing sites are mostly clade-specific. Finally, we observe that chloroplast RNA editing appears to be completely absent in horsetails (Equisetales), the sister clade of all other monilophytes.

**Conclusions:**

C-to-U and U-to-C RNA editing in fern chloroplasts and mitochondria follow disinct evolutionary pathways that are surprisingly different from what has previously been found in flowering plants. The results call for careful differentiation of the two types of RNA editing in the two endosymbiotic organelles in comparative evolutionary studies.

**Electronic supplementary material:**

The online version of this article (doi:10.1186/s12862-016-0707-z) contains supplementary material, which is available to authorized users.

## Background

RNA editing that converts specific cytidines into uridines in chloroplast and mitochondrial transcripts is nearly omnipresent among land plants [1–4]. As an apparently unique exception among plants, the marchantiid (complex-thalloid) liverworts have secondarily lost RNA editing [5, 6]. In most cases, RNA editing affects organelle mRNAs, where evolutionarily conserved codons are restored. However, numerous examples have also been found for RNA editing in tRNAs [7–10], UTRs [11], rRNAs [12], and introns [13–16]. Editing in the non-coding RNAs is likewise considered to be essential for the correct biological function of the respective molecules by re-establishing necessary base-pairing in secondary or tertiary RNA structures [15, 16].

Whereas the C-to-U type of RNA editing is nearly ubiquitous among land plants, the reverse process, U-to-C editing, appears to be more restricted in occurrence. U-to-C editing is unequivocally present in hornworts [17–19] and in monilophyte organelles [15, 20, 21]. U-to-C editing occurs in at least some lycophytes, the quillworts (Isoetales) and the club mosses (Lycopodiales) [10, 22–24]. It is surprisingly absent in Selaginellales, the third lycophyte order, despite having record numbers of C-to-U editing in both mitochondria and chloroplasts [12, 16].

The monilophytes are a morphologically heterogenous group that include the true eusporangiate ferns (sporangia with multicellular walls), such as Ophioglossales (moonworts) and Marattiales, the Equisetales (horsetails), the Psilotales (whisk ferns), and the species-rich group of leptosporangiate ferns (sporangia with unicellular walls). Early studies with *rbcL*, *atpB*, *rps4*, and nuclear 18S rDNA demonstrated the monophyly of the monilophytes and placed them as the sister group to the spermatophytes (seed plants) [25, 26]. However, the early dichotomies in the phylogeny of the monilophytes affecting the eusporangiate lineages and the horsetails relative to the leptosporangiate ferns remained unresolved. A recent study combining chloroplast loci *atpA*, *atpB*, *rbcL*, *rps4,* and *matK* with the mitochondrial loci *nad2*, *nad5*, *atp1,* and *rpl2* confidently placed the horsetails as sister group to all other monilophytes and Ophioglossales/Psilotales as the sister group to a joint clade of Marattiales and leptosporangiate ferns [27].

Previous studies on mitochondrial and chloroplast RNA editing in ferns already indicated highly differing frequencies of RNA editing in different fern taxa. In the chloroplasts of *Adiantum capillus-veneris* [28] and *Ophioglossum californicum* [29] RNA editing is abundant (315/35 and 297/3 C-to-U/U-to-C editing sites), whereas in *Psilotum nudum* only 27 C-to-U and no U-to-C editing sites were detected [29]. In the absence of a complete monilophyte mitochondrial genome, all mitochondrial RNA editing analyses in ferns are restricted to individual loci [15, 20, 21, 30].

The now available backbone phylogeny of monilophytes allows for phylogenetic insights into the evolution of C-to-U and U-to-C RNA editing among ferns, thus complementing similar studies restricted to C-to-U editing in flowering plants. To this end, we analyzed RNA editing in a phylogenetically wide sampling of monilophytes for four selected mitochondrial loci and for all available chloroplast genomic data. In contrast to an overall loss of RNA editing in angiosperms, we observed (i) an increase of RNA editing in monilophytes after the diversification of later emerging lineages, (ii) a mostly uncoupled evolution of editing frequencies in chloroplasts and mitochondria, and (iii) a largely uncoupled evolution of C-to-U and U-to-C editing. In some taxa, such as the order Schizaeales, U-to-C editing even exceeds the canonical C-to-U editing. Additionally, a complete chloroplast transcriptome analysis of the horsetail *Equisetum hyemale* confirmed our assumptions of total absence of RNA editing.

## Results

### Mitochondrial RNA editing in monilophytes

**Fig. 1:**
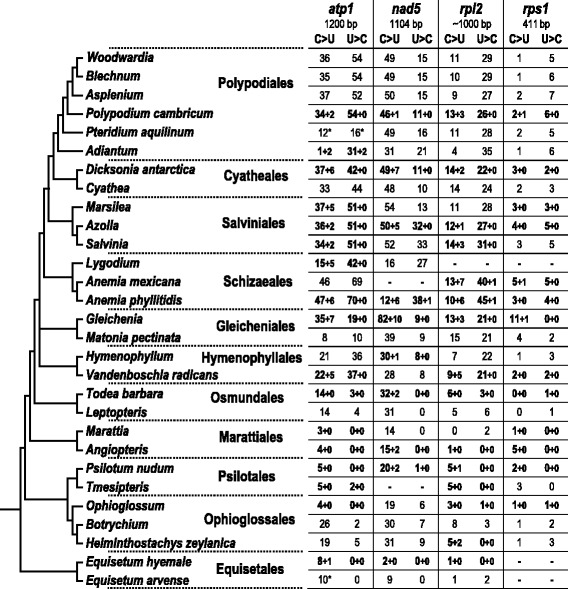
Mitochondrial RNA editing in a broad sampling of monilophyte taxa. The cladogram on the left is based on recent phylogenetic insights [27]. Experimentally verified (bold) and predicted (non-bold) RNA editing sites in the four mitochondrial loci *atp1*, *nad5*, *rpl2,* and *rps1* are shown. Predictions of RNA editing sites were done with PREPACT [70] as described under methods. Numbers behind the plus (+) signs indicate additional unpredictable silent edits identified in the cDNA sequences. Hyphens (−) indicate lacking data. Editing site numbers marked with an asterisk (*) are derived from shorter amplicon sequences. Amplicon lengths in *rpl2* vary in Equisetales, Ophioglossales, Psilotales and Marattiales owing to a hypervariable region in the first exon [27]

Our mitochondrial RNA editing analysis was based on four genes, which were previously included for phylogenetic studies in wide samplings of monilophyte taxa: *atp1, nad5, rpl2,* and *rps1* [21, 27, 31, 32]. Initial predictions were verified by cDNA analyses for some taxa. Since all four mitochondrial loci contain introns in most monilophyte taxa (atp1i361g2, nad5i1242g2, rpl2i846g2, rps1i25g2), spliced (and RNA edited) cDNAs could be identified by their smaller RT-PCR product sizes relative to the DNA-derived products. Details of taxon sampling and database accessions of the cDNA sequences are outlined in Additional file 1: Table S1. Results are summarized along the phylogeny of the taxa under investigation in Fig. 1.

Overall, the four different mitochondrial loci reflect comparable frequencies of RNA editing for each individual taxon (Fig. 1). However, the genus *Gleichenia* and *Anemia phyllitidis* are exceptions with unusual high numbers of RNA editing. *Gleichenia* has higher editing than all other examined taxa in two loci, the 1104 bp amplicon of the *nad5* gene (92 C-to-U and 9 U-to-C edits) and the 411 bp amplicon of the *rps1* gene (12 C-to-U edits). *A. phyllitidis* shows highest editing numbers in the other two loci, the *rpl2* gene (16 C-to-U and 46 U-to-C edits) and the *atp1* gene (53 C-to-U and 70 U-to-C edits).

**Fig. 2:**
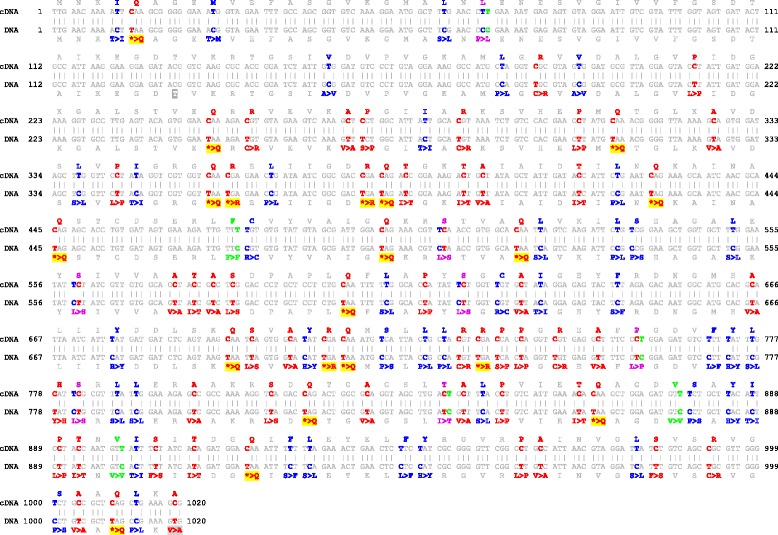
RNA editing in the mitochondrial *atp1* gene of *Anemia phyllitidis* (Schizaeales). Reverse U-to-C RNA editing (red) exceeds conventional C-to-U editing (blue). 123 editing sites were detected in the 1020 bp cDNA sequence. Green indicates silent edits and purple indicates multiple edits with more than one edit affecting an individual codon. The sequence display was obtained with the cDNA analysis mode of PREPACT [70]. Removal of stop codons is highlighted with yellow background shading. False negative and false positive predictions are shaded in light and dark gray, respectively

The *atp1* locus in *A. phyllitidis* has the most U-to-C editing and it was used for an exemplary outline of all 123 observed RNA editing events (Fig. 2). Reverse U-to-C editing includes the removal of 20 genomic stop codons. In comparison to the initial prediction by PREPACT, we detected six additional silent editing sites by RT-PCR; all of these are in 3rd codon positions that leave the encoded amino acids unchanged and hence are unpredictable. Interestingly, despite the overall dominance of U-to-C editing, all of these six silent editing sites are C-to-U conversions. Moreover, all sites of silent editing are located in immediate neighbourhood to non-silent edits, reminding of similar observations in the lycophyte *Selaginella uncinata* [16] where such sites have been termed “NESIs” (for neighbouring silents).

Of the remaining 117 non-silent edit sites, 115 were initially correctly predicted by PREPACT with other monilophyte cDNAs as references. One predicted candidate editing event (atp1eU134TM) remained unconfirmed in the *A. phyllitidis* cDNA. This false positive candidate site was also predicted for *Lygodium japonicum* but likewise remained unconfirmed. Accordingly, the overall number of 116 predicted events was very close to the actual number of verified 117 non-silent edit sites. Therefore, also editing predictions for other monilophyte taxa could be considered as trustworthy (Fig. 1).

Contrary to the high levels of RNA editing in *Anemia* or *Gleichenia*, we observed very low numbers of editing in the early-branching eusporangiate fern lineages and the horsetails (Equisetales). In contrast to *Anemia* and *Gleichenia* that each feature more than 200 editing sites in the four sampled mitochondrial loci, only 27 editing sites were found in the same four genes of *Angiopteris evecta*.

Other than overall high RNA editing numbers, *G. dicarpa* also shows widely divergent ratios of C-to-U vs. U-to-C editing ranging from 12:0 in *rps1* to 19:21 in *rpl2*. The latter example is the earliest emerging case of U-to-C editing exceeding C-to-U editing in our phylogenetic sampling (Fig. 1). The trend of U-to-C editing surpassing the canonical C-to-U editing becomes more pronounced in the later branching lineages, reaching a peak with an overall dominance of U-to-C over C-to-U editing at a ratio of 159:93 in *A. phyllitidis* (Schizaeales). While fewer U-to-C RNA editing sites were predicted (and confirmed) for the eusporangiate lineages Psilotales, Ophioglossales, and the Osmundales as the earliest-branching leptosporangiate lineage, we failed to find any evidence for U-to-C editing in Equisetales and Marattiales.

The ratio of C-to-U and U-to-C RNA editing becomes more balanced in the later emerging water ferns (Salviniales), tree ferns (Cyatheales) and in the large order of Polypodiales, which contains most of the extant fern diversity [33]. Resolving the phylogeny of Cyatheales and Salviniales relative to the Polypodiales has been demanding, likely owing to the extremely divergent generation times resulting in very short branch lengths of the tree ferns [34] as opposed to the long branches of the water ferns. Noteworthy are the very similar frequencies of RNA editing of both types in these two morphologically and developmentally extremely divergent fern groups (Fig. 1).

Our extended data set of 1794 mitochondrial editing sites identified by cDNA analysis (Fig. 1) revealed a strong bias of silent editing when the two types of pyrimidine conversions are compared. Out of altogether 960 documented events of C-to-U editing, 114 are silent (11.9 %). In contrast, only 7 of 834 identified sites of U-to-C editing (0.8 %) are silent.

### Comprehensive analysis of the *Equisetum hyemale* chloroplast transcriptome

In the course of our studies we observed very low numbers of predicted chloroplast RNA editing in the *Equisetum hyemale* chloroplast genome (accession number KC117177) [35]. No in-frame stop codons are present in the protein coding genes, which are a good indicator for reverse editing. However, a conserved putative start codon in the *accD* gene is ACG, which may be subject to C-to-U editing. In order to investigate the issue, we performed an exhaustive cDNA analysis of the *Equisetum hyemale* chloroplast coding regions. All annotated introns are spliced out correctly and all negative controls did not show any traces of a potential contamination of genomic DNA. However, we did not find a single RNA editing site in any protein coding gene. Even the creation of the putative AUG start codon by C-to-U editing in *accD* could not verified. We therefore conclude that the *Equisetum hyemale* chloroplast is completely devoid of RNA editing.

### Comparison of mitochondrial and chloroplastid RNA editing

For the comparison of mitochondrial and chloroplast RNA editing frequencies we investigated all available complete monilophyte chloroplast genomes. Extensive chloroplast editome analyses have previously been done for *A. capillus-veneris* [28], *O. californicum* and *P. nudum* [29] and are valuable additional references for predictions of RNA editing in the chloroplast genomes of *Alsophila spinulosa* [36], *Diplopterygium glaucum*, *Osmundastrum cinnamomeum* [37], *Angiopteris evecta* [38], *Marsilea crenata* and *Lygodium japonicum* [39].

**Fig. 3:**
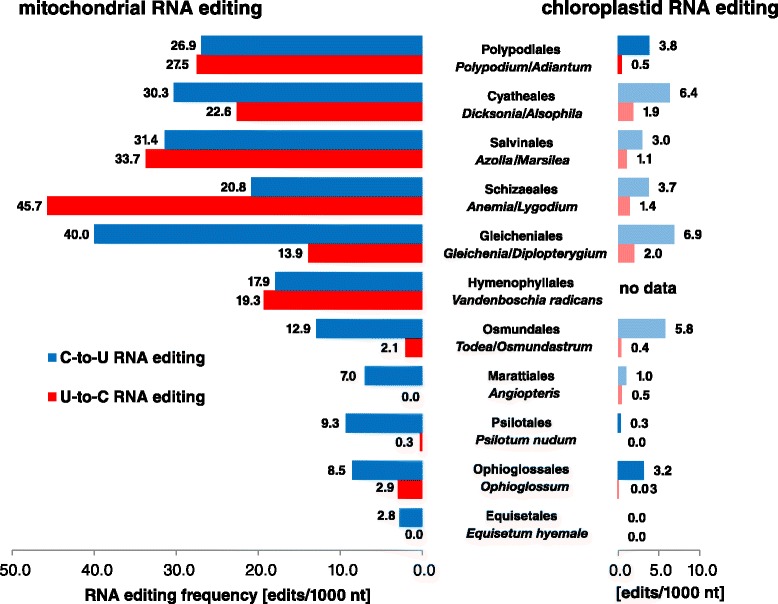
Comparison of mitochondrial (left) and chloroplast (right) RNA editing frequencies. Chloroplast RNA editing frequencies for *Adiantum capillus-veneris* [28], *Psilotum nudum* and *Ophioglossum californicum* [29] are derived from the respective publications. Editing analysis for *Equisetum hyemale* was done in this study. All other chloroplastid RNA editing frequencies are predicted (lighter colors) with the help of PREPACT [70] for complete plastome sequences available. Mitochondrial RNA editing frequencies include confirmed editing sites from the genes *atp1*, *rpl2*, *rps1*, and *nad5*

All editing frequencies were calculated as numbers of editing sites per 1000 nucleotides of coding sequence (Fig. 3). Like in other land plant clades, mitochondrial RNA editing frequencies generally exceed chloroplast RNA editing significantly. For C-to-U editing the mitochondria-to-chloroplast bias is in a range of 2.5-fold in Ophioglossales or Osmundales up to more than tenfold in Gleicheniales. However, this organelle bias is much more dramatic for U-to-C editing frequencies in mitochondria that are more than 30-fold the rate than in chloroplasts, e.g. in Polypodiales or Schizaeales.

We sampled the chloroplast genes *ndhF*, *chlB* and *chlL* to test for the low numbers of predicted RNA editing in *Angiopteris evecta* for comparison to the low levels of mitochondrial editing we identified (Fig. 1). Indeed, we identified only seven predicted C-to-U editing sites (chlBeU1268SL, chlLeU317SL, ndhFeU296SL, ndhFeU431PL, ndhFeU980PL, ndhFeU1001PL and ndhFeU1028SL) in the respective amplicons. Of these, only chlLeU317SL had previously been reported as a monilophyte editing site, in *Adiantum capillus-veneris* [28]. The *chlB* edit in *Angiopteris* is exclusively shared with the lycophyte *Selaginella uncinata* [16] and edit ndhFeU1001PL is shared with the basal angiosperm *Amborella* [40] and the hornwort *Anthoceros angustus* [19]. In contrast to the confirmed C-to-U editing sites, none of three predicted U-to-C edits (chlBeC923VA, ndhFeC826FL, ndhFeC847FL) were confirmed.

### Conservation of editing sites in mitochondria and chloroplasts

**Fig. 4:**
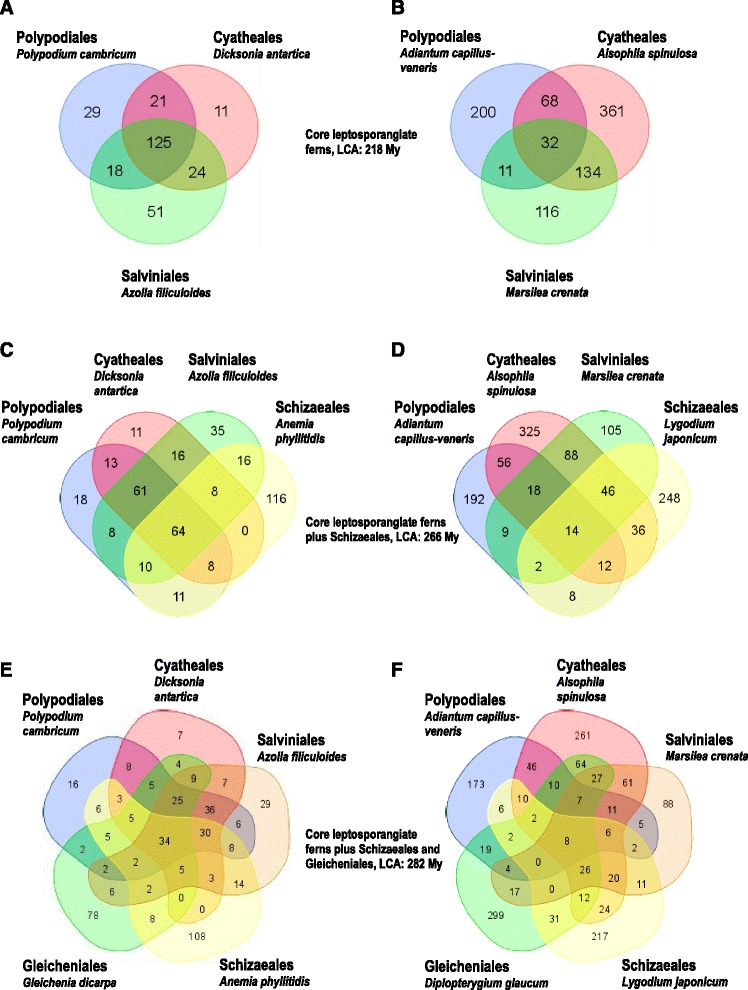
Venn diagrams of organelle RNA editing sites in leptosporangiate ferns. Non-silent editing sites of the three core leptosporangiate fern orders in mitochondria (**a**) and chloroplasts (**b**), of core leptosporangiate ferns and Schizaeales in mitochondria (**c**) and chloroplasts (**d**) and additionally including Gleicheniales to compare shared and unique mitochondrial (**e**) and chloroplast (**f**) editing sites. The approximate ages of the last common ancestors (LCAs) according to [72] are indicated. Venn diagrams were created using the online tool available under http://bioinformatics.psb.ugent.be/webtools/Venn/

We finally investigated to which extent individual mitochondrial and chloroplast RNA editing sites are conserved between different clades. Of altogether 279 non-silent mitochondrial editing sites present in the four mitochondrial genes of *Polypodium cambricum*, *Dicksonia antarctica* and *Azolla filiculoides* 45 % (125) are shared by all three species, indicating shared ancestry from a common ancestor of polypods, water ferns and tree ferns (Fig. 4a). An entirely different picture emerges for chloroplast RNA editing in the three orders (Fig. 4b). Of altogether 922 unique candidate edits only 3.5 % (32) are shared between the three representatives of Polypodiales, Cyatheales and Salviniales, respectively. The vast majority of chloroplast editing sites is unique to individual taxa, most prominently the 361 candidate sites of editing in *Alsophila* (Fig. 4b).

This discrepancy in the evolution of mitochondrial vs. chloroplast editing patterns is still confirmed when taxa of the heavy-editing Schizaeales (Figs. 4c, d) and Gleicheniales (Figs. 4e, f) are included. Although Schizaeales and Gleicheniales feature large numbers of unique mitochondrial editing sites, a high number of edits (64 and 34, respectively) are shared between all four or five orders (Figs. 4c and e). In contrast, of more than 1,000 chloroplast editing sites only 8 are shared among the representatives of all five clades, respectively.

## Discussion

Phylogenetic studies of plant organelle RNA editing have so far focused on flowering plants for several obvious reasons [40–43]. First, angiosperms contain most plant model taxa like *Arabidopsis*, rice, pea, wheat or tobacco where RNA editing has been studied extensively. Second, much more organelle genome information is available for comparative studies among flowering plants than for other plant clades. Finally, a very a good phylogenetic framework has been available for angiosperms since a couple of years.

Monilophytes, the sister group to seed plants (spermatophytes), are particularly interesting for comparative studies of organelle RNA editing evolution for two main reasons. First, the evolution of editing spans a much larger time frame since the monilophyte clade is about three times as old as the angiosperms. Second, the plant-typical C-to-U-type of RNA editing is accompanied by U-to-C editing in the reverse direction in monilophytes. Moreover, a well-supported backbone phylogeny of ferns has been obtained very recently [27], now offering the opportunity to trace the evolution of chloroplast and mitochondrial RNA editing of both types. We here present such a side-by-side study on organelle RNA editing in monilophytes paying particular attention to consider chloroplast vs. mitochondrial editing on the one hand and C-to-U vs. U-to-C editing on the other hand separately.

Several interesting observations emerge. Our analysis shows a high variance of RNA editing in monilophytes. There is a clear trend towards more editing in the more diversified and species-rich orders of the leptosporangiate ferns compared to the basal eusporangiate fern lineages. This is in full contrast to the large-scale evolution of RNA editing in angiosperms, where RNA editing is frequent in early lineages but significantly lower in later emerging lineages, both in mitochondria [44–46] and in chloroplasts [40].

Despite extreme differences in morphology and development, the three extant lineages of crown leptosporangiates, the Polypodiales, the Cyatheales (tree ferns) and the Salviniales (water ferns) show comparable mitochondrial RNA editing frequencies of ca. 30 editing sites per kb both for C-to-U and U-to-C editing (Fig. 3). Peak mitochondrial editing frequencies, however, occur in earlier-branching leptosporangiate orders with C-to-U editing reaching a top value at 40 sites per kb in *Gleichenia* and U-to-C editing with more than 45 events per kb in *Anemia*.

Particularly noteworthy is the finding that in *Anemia phyllitidis* more than two thirds of all editing sites are of the U-to-C type. This strong dominance of reverse over the canonical C-to-U editing has to our knowledge never been observed before in a plant organelle. Intriguingly, we find that such a dominance of reverse U-to-C editing is apparently restricted to the mitochondrial lineage. Even in the case of the Schizaeales, the fraction of reverse editing in the chloroplast amounts to only 30 % of all candidate sites. Certainly, the different genera analyzed in our estimates of chloroplast vs. mitochondrial RNA editing are a remaining caveat.

Comparable numbers of co-existing U-to-C and C-to-U editing have previously been found (or predicted) in hornworts [17–19]. Hornworts (Anthocerotophyta) obviously are another highly interesting plant clade for comparative studies of the different types of editing in the future. However, the current scarcity of organelle sequence data (in particular plastome sequences) and the low numbers of extant hornwort taxa impede such analyses.

Other than the highly variable numbers of C-to-U and U-to-C editing in the two endosymbiotic organelles across monilophyte diversity, the entirely different patterns of conservation of individual editing sites are striking. Only 3.5 % of chloroplast RNA editing sites are shared between the representatives of Salviniales, Cyatheales and Polypodiales (Fig. 4b). In contrast a full 45 % of mitochondrial RNA editing sites are shared between the representatives of the three orders (Fig. 4a), indicating shared ancestry of those sites from their last common ancestor living approximately 218 million years ago. Similar pictures emerge when representatives of the Schizaeales and Gleicheniales are serially included for comparison of editing site conservations in the ancestors living approximately 266 or 282 million years ago, respectively. However, the numbers of shared sites also decline very quickly for the mitochondrial comparisons, very likely owing to dramatic changes in the organelle editomes with numerous gains and losses in that period of monilophyte evolution. Both the individual RNA editing sites as well as their total numbers become more conserved in the mitochondrial lineage with the LCA of Cyatheales, Polypodiales and Salviniales. No similar observation can be made for the chloroplast lineage, however, indicating a highly dynamic gain and loss scenario in the chloroplast editomes. Again, as a caveat, denser sampling of taxa in both organelles will be needed in the future to evaluate these insights.

Gains and losses of organelle RNA editing sites are expected to be accompanied by corresponding changes in the nuclear-encoded co-factors addressing individual RNA editing sites in chloroplast or mitochondria. Key editing factors are unique RNA-binding pentatricopeptide repeat (PPR) proteins of the so-called “PLS-type”, which are particularly abundant in the plant lineage [47–49]. The dynamic gain and loss phases of organelle editing sites in monilophytes and in particular in the leptosporangiate ferns are expected to be accompanied by a massive gain, loss, or re-assignments of the nuclear encoded PLS-type PPR proteins. Unfortunately, ferns are notorious for their large, polyploid and complex genomes [50–52]. Hitherto available monilophyte transcriptome data, e.g. in the OneKP project [53] are as yet of insufficient quality and preclude to give good estimates on the complexity and diversity of PPR gene families or to make RNA targeting prognoses for individual members based on the recently deduced PPR-RNA binding code [54, 55]. Accordingly, no monilophyte sample has as yet been included in a very recent novel approach to comprehensively identify and distinguish PPR proteins in available genomic data [56]. This may hopefully change in the near future. A recent genome project sequencing the nuclear genome of *Azolla filiculoides* is ongoing and the nuclear genome of *Ceratopteris richardii* is proposed as a possible project [57].

Other than their tremendous diversity of editing patterns and the simultaneous existence of reverse U-to-C editing, ferns offer yet another feature distinguishing them from flowering plants with regard to organelle RNA editing. RNA editing in angiosperms is affected by additional protein factors called multiple organelle RNA editing factors (MORFs) or RNA editing factor interacting proteins (RIPs) [58–61], which assemble together with PPR proteins as complex editosomes on the target RNAs. No evidence for MORFs/RIPs is identified in the hitherto available fern genome or transcriptome data, very much like in the more ancient bryophyte or lycophyte lineages. Hence, the monilophyte clade may have retained a more ancient and simple RNA editing machinery devoid of helper proteins like MORFs/RIPs.

Most importantly, any forthcoming fern genome data will help to elucidate the yet enigmatic biochemical machinery of reverse U-to-C editing. The DYW domain with cytidine deaminase similarity present at the carboxyterminus of many PLS-type RNA editing factors is the bona fide candidate to perform the deamination of cytidine to uridine [62]. No convincing protein candidate has a yet been proposed for the reverse reaction evidently requiring an amino group donor as a co-substrate for the amination of uridine. Any proteins proposed to be involved in U-to-C editing should be correspondingly diverse in taxa for which we here demonstrate a high proportion in this type of editing. Of particular interest in this respect is the much lower amount of silent editing for U-to-C conversions (0.8 %) in comparison to the phylogenetically more widely distributed C-to-U editing (11.9 %). A similar bias has previously emerged for silent editing in mitochondria of *Isoetes engelmannii* [9]. Whether these findings may indicate a higher fidelity of sequence recognition specificity of the yet elusive U-to-C editing factors remains to be seen.

The recent editing analysis of the chloroplast genomes of *Ophioglossum californicum* and *Psilotum nudum* assumed that reverse editing is entirely lost in *Psilotum* and that it may also be lost in *Equisetum*, *Angiopteris* and *Osmundastrum* based on the absence of in-frame stop codons in chloroplastid genes [29]. Here, we found that RNA editing in both organelles evolves independently and show experimental evidence for reverse editing in *Psilotum nudum* mitochondria (Fig. 1). The hypothesis of a complete loss of reverse editing in *Angiopteris* and *Equisetum*, and possibly in the orders Marattiales and Equisetales altogether, is supported by our study, however. Similarly, no evidence for reverse RNA editing had previously been found in the study of other mitochondrial genes in *Equisetum* [30].

Moreover, we could not find any evidence for RNA editing at all in our extensive transcriptome study of the chloroplast genome of *Equisetum hyemale*. To our knowledge this would be the first example of RNA editing documented in only one of the two endosymbiotic organelles. Extrapolating from the only 12 mitochondrial editing sites identified in the three amplicons investigated here (Fig. 1) one can assume that, depending on its total mitochondrial gene complement, *Equisetum hyemale* may have only around 100 mitochondrial editing sites. Even in the case of the model moss *Physcomitrella patens* with only 11 mitochondrial editing sites [63, 64], two editing sites exist in the chloroplast [65, 66]. Similarly reduced RNA editing is observed for sister taxa in other Funariaceae mosses, too [67].

The overall low amount of RNA editing in the early-branching eusporangiate lineages, the complete absence of chloroplast editing in *Equisetum hyemale* and the likely absence of reverse editing altogether in Marattiales and Equisetales may indicate that the last common ancestor of monilophytes had very low amounts of RNA editing in general and reverse U-to-C editing in particular. However, we are very reluctant to come to this conclusion given that members of the lycophytes, the sister lineages to euphyllophytes (comprising monilophytes and spermatophytes), feature highly frequent and highly diverse RNA editing in both organelles, exclusively of the C-to-U type in Selaginellales [12, 16] and in both direction of pyrimidine exchange in the Isoetales [10]. Since fern mitochondrial RNA editing proved to be more deeply conserved we checked for its conservation among the lycophytes (Additional file 2: Figure S1). This comparison shows that the overwhelming majority of editing sites in the high-level editing lycophytes *Isoetes* and *Selaginella* are taxon-specific, notably also in comparison to the low-editing, early-branching lycophyte *Phlegmariurus* (Additional file 2: Figure S1B), making independent gains of most editing sites in *Isoetes* and *Selaginella* more likely.

One way or the other, the monilophytes have experienced dramatic changes in their editomes. Our results warrant for differentiated considerations of plant organelle RNA editing, both with respect to mitochondria vs. chloroplasts and with respect to C-to-U vs. U-to-C exchanges, for which we here show independent patterns of evolution. The most intriguing open question concerns the evolutionary forces driving the massive increases of C-to-U but notably also U-to-C RNA editing during the diversification of the early leptosporangiate lineages.

## Conclusion

This study reports a broad-scale evolutionary comparative analysis of C-to-U and U-to-C RNA editing in the two endosymbiotic organelles of monilophytes. We find that mitochondrial RNA editing is highly diverse in monilophytes, including particularly low editing rates in early vs. late branching lineages, which is in full contrast to the evolutionary patterns previously observed among flowering plants. The reverse type of U-to-C RNA editing appears to be completely absent in Equisetales (horsetails) and in Marattiales. Within the leptosporangiate ferns, however, RNA editing of both types is highly abundant with record amounts of reverse editing in Schizaeales. Mitochondrial RNA editing sites in the leptosporangiate ferns are strikingly conserved in contrast to RNA editing in the chloroplasts. Hence, C-to-U and U-to-C RNA editing is evolving independently in the two organelles and the results call for careful differentiation between the different types of RNA editing in mitochondria and chloroplasts. Its great variability in the fern organelles promises an interesting field of co-evolution of C-to-U and U-to-C editing sites and their hitherto unknown nuclear-encoded specificity factors. Importantly, the biochemical mechanisms of “reverse” U-to-C editing, evolutionarily much more restricted than the more widespread C-to-U editing both within and outside of the plant kingdom [2], are completely unknown at present. Whereas cytidine deaminases are key to the latter editing type, a yet completely enigmatic transamination process involving an unknown amino-group donor must be postulated for the former. We believe that the here documented diversity of editing in both directions among monilophytes will ultimately help to identify the U-to-C editing factors and explain their unique features such as the here identified low amount of accompanying “superfluous” silent editing possibly indicating a higher degree of target specificity than the factors performing C-to-U editing.

## Methods

### Plant material and molecular work

Plant material was obtained from the Botanic Garden Bonn. Nucleic acids were isolated according to the CTAB method [68] followed by DNA digestion with DNAse I (Thermo Scientific/Fermentas). Total RNA was reverse transcribed into single-stranded cDNA with the RevertAid First Strand cDNA Synthesis Kit (Thermo Scientific/Fermentas). Priming was performed with random hexamer primers (Roth). Subsequent RT-PCRs with Go-Taq (Promega) and/or Q5 Polymerase (New England Biolabs) were done with primers from [21, 27, 31]. For the transcriptome analysis of the *Equisetum hyemale* chloroplast primers binding in the UTRs of single or polycistronic genes were used. As a negative control samples without reverse transcriptase treatment were used to test for remnants of genomic DNA.

RT-PCR products were separated by agarose gel electrophoresis and recovered by the NucleoSpin Extract II Kit (Macherey Nagel). Gel-eluted products were either sequenced directly or after cloning into the pGEM-T Easy vector (Promega) and amplification in *Escherichia coli*. Sequencing was done by Macrogen Europe (Amsterdam, NL). New cDNA sequences were submitted to GenBank (see Additional file 1: Table S1). Sequences were analyzed with MEGA 5.05 [69] and aligned using the implemented ClustalW algorithm.

### RNA editing analysis

For each of the four investigated mitochondrial loci (*atp1, nad5, rpl2, rps1*), cDNA sequences were determined for at least 10 fern taxa (Fig. 1, Additional file 1: Table S1). The cDNA sequences were employed as references to predict mitochondrial RNA editing sites in the respective other monilophyte taxa for which we did not generate cDNA sequences using the alignment prediction tool of PREPACT [70]. Sites were counted as candidate editing sites when predicted by at least 80 % of the references.

Chloroplast RNA editing sites were predicted with the BLASTX prediction tool of PREPACT. Again, a threshold for prediction of 80 % and ten selected reference sequences were used: *Adiantum capillus-veneris*, *Amborella trichopoda, Arabidopsis thaliana*, *Chara vulgaris*, *Equisetum hyemale*, *Marchantia polymorpha*, *Ophioglossum californicum, Pellia endiviifolia*, *Physcomitrella patens* and *Psilotum nudum*. All editing sites were labeled according to a previously published nomenclature proposal [64, 71] using the name of the gene, type of editing, the nucleotide position and the respective amino acid change.

## Additional files

Additional file 1: Table S1. Monilophyte taxon sampling. Database accessions are given for the sequences of the 4 loci investigated for RNA editing analysis. Accession numbers in bold indicate new sequences obtained in this study (n. d., no data). Accessions labelled with the degree symbol (°) are from closely related species of the same genus. (DOCX 18 kb) Additional file 2: Figure S1. Comparison of mitochondrial RNA editing sites between monilophytes and lycophytes. The experimentally confirmed editing sites in the genes *atp1* and *nad5* of the monilophyte species *Polypodium cambricum*, *Dicksonia antarctica* and *Azolla filiculoides* are compared with the respective editing sites deposited in NCBI from the lycophytes *Isoetes engelmannii* and *Selaginella moellendorffii*. Only 16 edits are shared between all five taxa. Most of the edits from the two lycophytes are unique to either one species or are shared between the two lycophytes and are therefore most likely independent gains. For the basal lycophyte *Phlegmariurus squarrosus* only 14 edits (all of the C-to-U type) are found in our cDNA analysis. Three of these edits are shared between all three lycophytes and six between *Phlegmariurus* and *Isoetes*. (DOCX 127 kb)
