# Supplementary material for: Reverse U-to-C editing exceeds C-to-U RNA editing in some ferns – a monilophyte-wide comparison of chloroplast and mitochondrial RNA editing suggests independent evolution of the two processes in both organelles
Source: BMC Evol Biol. 2016 Jun 21;16:134. doi: 10.1186/s12862-016-0707-z (PMC4915041; doi:10.1186/s12862-016-0707-z)
Supplement: Additional file 1: Table S1. — Monilophyte taxon sampling. Database accessions are given for the sequences of the 4 loci investigated for RNA editing analysis. Accession numbers in bold indicate new sequences obtained in this study (n. d., no data). Accessions labelled with the degree symbol (°) are from closely related species of the same genus. (DOCX 18 kb) [file 12862_2016_707_MOESM1_ESM.docx]

**Supplementary Table 1. Monilophyte taxon sampling.** Database accessions are given for the sequences of the 4 loci investigated for RNA editing analysis. Accession numbers in bold indicate new sequences obtained in this study (n. d., no data). Accessions labelled with the degree symbol (°) are from closely related species of the same genus.

| **Monilophytes** | ***atp1*** | ***atp1* cDNA** | ***nad5*** | ***nad5* cDNA** | ***rpl2*** | ***rpl2* cDNA** | ***rps1*** | ***rps1* cDNA** |
| --- | --- | --- | --- | --- | --- | --- | --- | --- |
| ***Adiantum capillus-veneris*** | DQ110142 | **KU744738** | KJ944536 | n. d. | KP757852 | n. d. | KU352806 | n. d. |
| ***Anemia mexicana*** | KJ944570 | n. d. | n. d. | n. d. | KJ944516 | **KU744725** | KU352822 | **KU744734** |
| ***Anemia phyllitidis*** | KJ944571 | **KU744697** | KJ944537 | **KU744714** | KJ944517 | **KU744726** | KU352823 | **KU744735** |
| ***Angiopteris madagascariensis*** | KJ944577 | **KU744701** | KJ944542 | **KU744718°** | KJ944524 | **KU352844** | KU352833 | **KU352850** |
| ***Asplenium nidus*** | KJ944566 | n. d. | AJ130738 | n. d. | KJ944513 | n. d. | KU352807 | n. d. |
| ***Azolla filiculoides*** | KJ944561 | **KU744696** | AJ130739 | **KU744713** | KJ944507 | **KU744723** | KU352817 | **KU744733** |
| ***Blechnum gibbum*** | KP757842 | n. d. | AJ130740° | n. d. | KP757853 | n. d. | KU352808 | n. d. |
| ***Botrychium lunaria*** | DQ110159° | n. d. | AJ130742 | n. d. | KJ944530 | n. d. | KU352835 | n. d. |
| ***Cyathea dealbata*** | KP757843° | n. d. | KJ944533 | n. d. | KJ944511 | n. d. | KU352814 | n. d. |
| ***Dicksonia antarctica*** | AJ548853 | **KU744695** | AJ130745 | **KU744712** | KJ944510 | **KU744722** | KU352815 | **KU744731** |
| ***Equisetum arvense*** | DQ646213 | **KU744710°** | AJ130749° | n. d. | KJ944532° | n. d. | n. d. | n. d. |
| ***Equisetum hyemale*** | KJ944851° | **KU744704** | AJ130748 | **KU744720** | KJ944531 | **KU352847** | n. d. | n. d. |
| ***Gleichenia dicarpa*** | KJ944572 | **KU744698** | KJ944538 | **KU744716** | KJ944518 | **KU352842** | KU352824 | **KU352848** |
| ***Helminthostachys zeylanica*** | DQ110149 | n. d. | KP757856 | n. d. | KJ944529 | **KU744729** | KU352836 | n. d. |
| ***Hymenophyllum trichomanoides*** | DQ646226° | n. d. | KJ944539° | **KU744715°** | KJ944521 | n. d. | KU352826 | n. d. |
| ***Leptopteris superba*** | KJ944576 | n. d. | KJ944541 | n. d. | KJ944523 | n. d. | KU352828 | n. d. |
| ***Lygodium japonicum*** | KP757845 | **KU744707** | AJ130750 | n. d. | n. d. | n. d. | n. d. | n. d. |
| ***Marattia laevis*** | KJ944578 | **KU744708** | KJ944543 | n. d. | KJ944525 | n. d. | KU352834 | **KU744737** |
| ***Marsilea drummondii*** | KJ944563 | **KU744706** | AJ131136 | n. d. | KJ944509° | n. d. | KU352818 | **KU744732** |
| ***Matonia pectinata*** | KJ944573 | n. d. | AJ131137 | n. d. | KJ944519 | n. d. | KU352825 | n. d. |
| ***Ophioglossum petiolatum*** | KJ944580° | **KU744702** | AJ131139° | n. d. | KJ944528 | **KU352845** | KU352838 | **KU352852** |
| ***Polypodium cambricum*** | KJ944565 | **KU744694** | KJ944534° | **KU744711** | KJ944512 | **KU744721** | KU352809 | **KU744730** |
| ***Pteridium aquilinum*** | AJ548852 | n. d. | KP757857 | n. d. | KP757854 | n. d. | KU352810 | n. d. |
| ***Psilotum nudum*** | AJ548873 | **KU744703** | AJ012794 | **KU744719** | KJ944526 | **KU352846** | KU352840 | **KU352850** |
| ***Salvinia molesta*** | KJ944562 | **KU744705** | AJ131147° | n. d. | KJ944508 | **KU744724** | KU352820 | n. d. |
| ***Tmesipteris elongata*** | KJ944579 | **KU744709** | n. d. | n. d. | KJ944527 | **KU744728** | KU352841 | n. d. |
| ***Todea barbara*** | KJ944575 | **KU744700** | KJ944540 | **KU744717** | KJ944522 | **KU352843** | KU352831 | **KU352849** |
| ***Vandenboschia radicans*** | KJ944574 | **KU744699** | AJ131145 | n. d. | KJ944520 | **KU744727** | KU352827 | **KU744736** |
| ***Woodwardia radicans*** | KJ944567 | n. d. | KJ944535 | n. d. | KJ944514 | n. d. | KU352812 | n. d. |
