# Supplementary material for: Reverse U-to-C editing exceeds C-to-U RNA editing in some ferns – a monilophyte-wide comparison of chloroplast and mitochondrial RNA editing suggests independent evolution of the two processes in both organelles
Source: BMC Evol Biol. 2016 Jun 21;16:134. doi: 10.1186/s12862-016-0707-z (PMC4915041; doi:10.1186/s12862-016-0707-z)
Supplement: Additional file 2: Figure S1. — Comparison of mitochondrial RNA editing sites between monilophytes and lycophytes. The experimentally confirmed editing sites in the genes atp1 and nad5 of the monilophyte species Polypodium cambricum, Dicksonia antarctica and Azolla filiculoides are compared with the respective editing sites deposited in NCBI from the lycophytes Isoetes engelmannii and Selaginella moellendorffii. Only 16 edits are shared between all five taxa. Most of the edits from the two lycophytes are unique to either one species or are shared between the two lycophytes and are therefore most likely independent gains. For the basal lycophyte Phlegmariurus squarrosus only 14 edits (all of the C-to-U type) are found in our cDNA analysis. Three of these edits are shared between all three lycophytes and six between Phlegmariurus and Isoetes. (DOCX 127 kb) [file 12862_2016_707_MOESM2_ESM.docx]

**B**

**A**

**Isoetales**

***Isoetes engelmannii***

**Isoetales**

***Isoetes engelmannii***

**Selaginellales**

***Selaginella moellendorffii***

**Selaginellales**

***Selaginella moellendorffii***

**
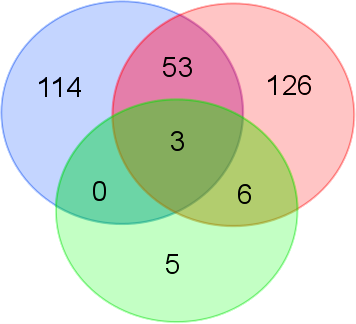

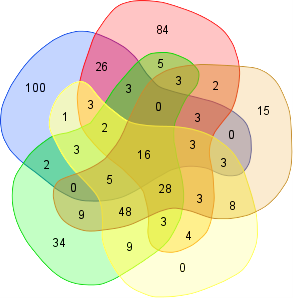
**

**Polypodiales**

***Polypodium***

***cambricum***

**Lycopodiales**

***Phlegmariurus squarrosus***

**Salviniales**

***Azolla filiculoides***

**Cyatheales**

***Dicksonia antartica***

**Supplementary Figure 1.** Comparison of mitochondrial RNA editing sites between monilophytes and lycophytes. Note that *rps1* and *rpl2* are not present in the lycophyte mtDNAs except for *rpl2* in *Phlegmariurus* *squarrosus*. The experimentally confirmed editing sites in the genes *atp1* and *nad5* of the monilophyte species *Polypodium* *cambricum*, *Dicksonia* *antarctica* and *Azolla* *filiculoides* are compared with those identified previously in the heavy-editing lycophytes *Isoetes* *engelmannii* and *Selaginella* *moellendorffii* (Grewe et al., 2011, Hecht et al., 2011). **A.** The majority of editing sites in the two lycophytes are unique to one species (100 or 84, respectively), some are shared between the two lycophytes (26), but only 16 edits are shared with all three fern taxa. **B.** For the low-editing lycophyte *Phlegmariurus* *squarrosus* representing the early-branching order Lycopodiales among the lycophytes, we identified only 14 edits in the two mitochondrial genes (Gerke et al., unpublished observations), of which only 3 are shared with *Isoetes* and *Selaginella*.
